# Larval nutrition-induced plasticity affects reproduction and gene expression of the ladybeetle, *Cryptolaemus montrouzieri*

**DOI:** 10.1186/s12862-015-0549-0

**Published:** 2015-12-08

**Authors:** Jiaqin Xie, Patrick De Clercq, Chang Pan, Haosen Li, Yuhong Zhang, Hong Pang

**Affiliations:** State Key Laboratory of Biocontrol, Key Laboratory of Biodiversity Dynamics and Conservation of Guangdong Higher Education Institute, College of Ecology and Evolution, Sun Yat-sen University, Guangzhou, 510275 China; Department of Crop Protection, Faculty of Bioscience Engineering, Ghent University, Ghent, B-9000 Belgium; Guangdong Entomological Institute, Guangzhou, 510260 China

**Keywords:** Food availability, *Cryptolaemus montrouzieri*, Developmental plasticity, Fitness, Gene expression

## Abstract

**Background:**

Organisms may develop into multiple phenotypes under different nutritional environments by developmental plasticity, whereas the potential costs and mechanisms of such plasticity are poorly understood. Here we examined the fitness and gene expression of nutrition-induced phenotypes in the ladybeetle, *Cryptolaemus montrouzieri* after having experienced varying larval food regimes*.*

**Results:**

We found that *C. montrouzieri* adults undergoing a variable larval food regime achieved a similar developmental time, survival, body mass and egg production as those undergoing a high larval food regime. The survival, developmental time, body mass and fecundity of the adults from a restricted larval food regime were inferior to those from the high and variable larval food regimes. However, the adults from this restricted larval food regime had a higher expression level of genes encoding immune- and antioxidant-related enzymes than those from the high and variable larval food regimes when exposed to starvation and pesticide conditions in adult life.

**Conclusions:**

These results suggest that larval food availability in *C. montrouzieri* not only triggers adult phenotypic differences but also affects reproduction and expression level of genes in adult life, indicating that the larval nutritional conditions can affect adult fitness and resistance to stressful conditions through developmental plasticity.

## Background

Developmental plasticity is an adaptive process that gives rise to multiple phenotypes under different environmental conditions [1–3]. Many factors may affect this process, including biotic (i.e. parasites, pathogens) and abiotic cues (temperature, humidity and photoperiod) [2, 4–6]. Organismal phenotypes adaptably vary in response to external environments, resulting in changes in body size, color or wing pattern [7–9]. Although the environment-induced traits are generally phenotypic variations that do not involve mutation or changes in DNA sequence, the plasticity plays crucial role in maintaining the adaptability of individuals to potential environmental fluctuations [1, 6, 10]. For example, the nutrition-induced horn dimorphism of male *Onthophagus taurus* scarabs enhances their mating success and reproduction under certain external conditions [11]; the larval diet regime is associated with the caste of honeybees [12]. Furthermore, studies have reported that under certain conditions, the environment-induced phenotypes are genetically fixed through genetic assimilation, suggesting that developmental plasticity may accelerate adaptive evolution [13].

To better match the phenotypes and selective environments, organisms adapt to the environmental conditions under which they live in by altering their behavior, physiology or morphology. The environmental conditions organisms experienced in their early life stages predict what conditions they will encounter in later life and allow them to adapt to such conditions by developmental plasticity [2, 14]. For instance, the nutrition-induced wing formation in the female pea aphid, *Acyrthosiphon pisum*, affects its dispersal in adult life [9]. However, the mechanisms of nutrition-induced developmental plasticity remain elusive [1, 15, 16]. A few studies report that hormones and DNA methylation affect the differentiation of adult phenotypes by regulating gene expression [17, 18]. More recently, it has been reported that cells also adapt their phenotypes by sensing the environmental conditions through a cell-intrinsic molecular mechanism by activating focal adhesion kinase (FAK, also known as PTK2), resulting in the adaptation of genes controlling membrane homeostasis [19]. Those studies have shed first light onto the molecular mechanism of developmental plasticity in response to external environments.

Larval nutritional conditions may affect adult reproductive performance and physiological functions in insects (e.g. metabolism, immune and antioxidant activities) [4, 20–24]. Whereas developmental plasticity has been noted to allow individuals to better respond to varying environmental conditions, less attention has been paid to the potential challenges of such plasticity on the fitness of the resulting phenotypes and to the effects on the expression levels of genes. In the present study, we examined the development, reproduction and gene expression of adults that experienced varying larval food regimes in the ladybeetle *C. montrouzieri*. The ladybeetle originates from Australia and has been introduced into at least 64 countries or regions around the world as a biological control agent [25, 26]. Its growth rate and phenotypes are largely nutrition-dependent, which makes the ladybeetle a suitable model to study nutrition-induced developmental plasticity and its effects on the fitness of the resulting phenotypes [21, 26].

We manipulated the food availability of the ladybeetle during its larval stages. *C. montrouzieri* larvae were subjected to high, variable and low food regimes, allowing them to grow at a normal, variable and restricted rate, respectively. Upon adult emergence, we examined the body mass, developmental time and survival of the ladybeetles from the different larval treatments. We then examined their reproductive performance (pre-oviposition period, fecundity, egg hatch) under an ad libitum food regime. Subsequently, we assessed the expression level of genes encoding immune- and antioxidant-related enzymes in adults under stressful conditions (i.e. starvation and pesticide exposure) using qPCR, and evaluated the starvation resistance of the adults from each larval food regime. The hypothesis was that if *C. montrouzieri* plastically developed in response to varying larval food regimes, their growth rate and adult phenotypes might be different. The resulting phenotypes from varying larval food regimes were expected to display different adult traits (e.g. reproductive output or gene expression).

## Methods

### Insect cultures

*C. montrouzieri* used in the experiments were obtained from a laboratory culture at Sun Yat-sen University, Guangzhou, China. The ladybeetles were reared on citrus mealybugs, *Planococcus citri* maintained on pumpkin fruits at ambient conditions (T = 25 ± 1 °C, RH = 60 ± 10 %). Prior to the experiments, 50 pairs of females and males were randomly collected from the stock culture and placed in individual Petri dishes (90 mm × 15 mm) to oviposit. We used cotton as an oviposition substrate and collected eggs from the third to seventh day of adult life. The eggs were allowed to hatch in a climate chamber at 25 ± 1 °C, 70 ± 5 % RH and a 14:10 h (L:D) photoperiod. Four days later, the emerging larvae were used in the experiment.

### Developmental plasticity

Newly hatched (<12 h) larvae of *C. montrouzieri* were transferred to Petri dish arenas (90 mm × 15 mm) and subjected to one of the three food regimes (n = 95, 95 and 105 for high, variable and restricted larval food regimes, respectively), simulating natural conditions of food abundance, variation in food supply and food scarcity. First and second instars of *C. montrouzieri* were maintained in groups of ten in each Petri dish; from the third instar on, each predator larva was isolated in an individual Petri dish. In the high food regime, ten *P. citri* (ca. 1.5 mm long) were supplied to each Petri dish and refreshed daily, allowing the ladybeetle to grow at normal rate. In the variable food regime, five *P. citri* larvae were supplied to each Petri dish and replaced every 48 h during the first eight days; from the ninth day on, food was supplied to the larvae in the same manner as in the high level food regime, allowing the individuals to experience compensatory growth [21]. Finally, in the low food regime, five *P. citri* larvae were supplied in each Petri dish and replaced every 48 h until the emergence of the adult ladybeetle, resulting in a restricted growth pattern. Thus, developmental plasticity yielded different phenotypes (i.e. restricted, compensatory and normal growth phenotypes) in response to varying larval food regimes. We assessed adult body mass within 48 h of adult emergence (n = 15 for each sex) using an electronic balance (Sartorius BSA124S, Germany, ± 0.1 mg). We also calculated the developmental time (from egg hatch to adult emergence) and survival rate of each group (the number of emerged adults out of the number of hatched larvae in each group).

### Reproductive performance

To examine the reproductive performance of the different resulting phenotypes, the emerging adult males and females from the same group were randomly paired (n = 15) and supplied ad libitum with *P. citri* from adult emergence. Each pair was placed in an individual 90 mm diameter Petri dish and maintained in a climate chamber set at 25 ± 1 °C, 70 ± 5 % RH and a 14:10 h (L:D) photoperiod. Their foods were refreshed and eggs were collected daily. The pre-oviposition period (from the time of pairing to first oviposition) was determined, and the numbers of deposited and hatched eggs were recorded within one month.

### Starvation resistance

Upon adult emergence, we evaluated the starvation resistance of the resulting adult phenotypes of *C. montrouzieri*. We examined the body mass change of female adults from the three larval treatments when experiencing a starvation period of five days (i.e. without food and water) versus ad libitum feeding on *P. citri*. First the initial body mass of the emerging female adults was recorded. Then, the females were placed into individual Petri dishes and exposed to the starvation or ad libitum food treatment in a climatic chamber at 25 ± 1 °C, 70 ± 5 % RH and a 14:10 h (L:D) photoperiod (n = 15). After 5 days, the final body mass of the adults was determined, and the mass change (△M) was calculated as (initial mass-final mass)/initial mass (%). Further, we randomly collected ten (five for each sex) *C. montrouzieri* adults from each larval food regime on the tenth day, i.e. when they were reproductively active, to assess their starvation resistance. For this purpose, the beetles were placed in individual Petri dishes and deprived of food and water at 25 ± 1 °C, 70 ± 5 % RH and a 14:10 h (L:D) photoperiod. Survival was monitored daily until all of the individuals had died.

### Gene expression

To evaluate the gene expression in the adult female resulting phenotypes of *C. montrouzieri*, quantitative real-time PCR (qPCR) was performed to estimate the expression of genes (i.e. lysozyme, acid phosphatase, phenol oxidase, peroxidase, HSP60 and carboxylesterase) under two types of stress conditions: (1) starvation for 24 h, (2) exposure to 4ul of the insecticide acetamiprid (16 ppm; Guoguang Agrochemical Co., Ltd, Sichuan, China) applied on the females’ pronotum using a micropipette. The selected enzymes have important functions in immune or antioxidant activities [27, 28]. Food scarcity has been reported to affect individual physiological functions, but less attention has been paid to the expression level of genes. Acetamiprid is a commonly used insecticide in agriculture and has previously been reported to be harmful to *C. montrouzieri* [29]. All adult females used in the experiment were randomly collected from the different phenotype groups on the tenth day of adult life. The extraction of RNA, transcription and qPCR amplification were carried out according to standard procedures [20, 22, 30]. Briefly, total RNA of adults was extracted using Trizol Total Isolation Kit (Invitrogen), and reverse transcription primed with oligo-dT was used to synthesize cDNA. Extractions of three individuals from each resulting phenotype after having undergone starvation or pesticide treatment were performed and two replicates of each extraction were used for qPCR. Relative transcript abundance was measured using qPCR on ABI STEPONE PLUS according to the manufacturer’s protocols for SYBR Green (by BGI-Tech, Shenzhen, China). The tubulin beta (BT) gene was selected as the reference gene. The gene primers and sequences used in RT-qPCR amplification are given in Table 1.

**Table 1:** Reference gene and gene sequences encoding immune and antioxidant enzymes used in qPCR

Genes	Sequences (5’-3’)	GenBank
Lysozyme	F-TATTCCACGCGACCAGTTGG	KT358925
	R-GGCGAGTCAGATTTGGAGCA	
Acid phosphatase	F-TACCGGAATGGACCCGTATG	KT363040
	R-TCGTAGAACCGTCCCAAGGA	
Pro-phenol oxidase	F-AAGGAGGAGACGAACTGGCC	KT363041
	R-CAATTGGCATGCAGTTCCTG	
Carboxylesterase	F-TCAAGGCCAGCTTCTGATGA	KT363042
	R-GAAGAAGTGGATCCGCGTCT	
Peroxidase	F-CAGCGAATCCCAGTTGGATG	KT363043
	R-TGGCTTCGAACACACGAGGT	
HSP60	F-GCCGTCGAGGAAGGTATCGT	KT363038
	R-ACACCGTTTGCTTGATCCGA	
Tubulin beta	F-CTCCGACGAACATGGAATCG	ADI24738.1
	R-TTGCCACCAGACGCTTCATT	

### Statistical analysis

Before analysis, all datasets were first tested for normality and homogeneity of variances by a Shapiro-Wilk test and Levene test, respectively. We used one-way analysis of variance (ANOVA) followed by Tukey tests to analyze the effect of larval food regimes (high, variable and low food regimes) on developmental time, adult body mass, egg hatch and body mass change. The egg production, pre-oviposition period and gene expression were tested using a Kruskal-Wallis test followed by Mann-Whitney *U* test due to the lack of normal distribution of the data. The survival rate was compared by a logistic regression, which is a Generalized Linear Model using probit link and a binomial error function. The significance level of all tests was set at *p* ≤ 0.05. All analyses were performed using SPSS 21 (IBM SPSS Statistical, Chicago, USA).

## Results

### Developmental plasticity

**Fig. 1:**
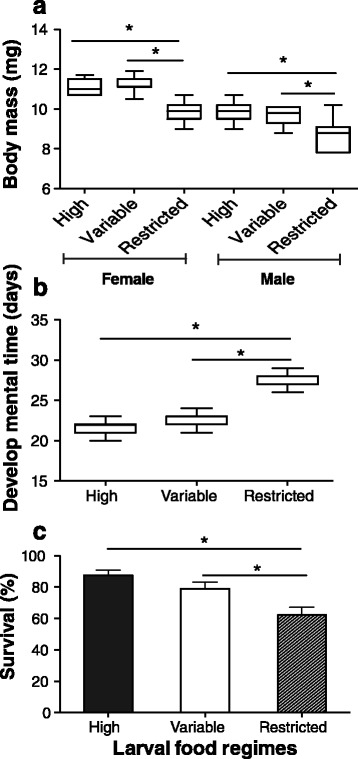
Effects of larval food regimes (high, variable and restricted) on development of *C. montrouzieri*. **a** Body mass of male and female adults at emergence; **b**, Larval and pupal developmental time; **c**, Survival from egg hatch to adult emergence. Asterisks (*) indicate significant differences. Error bars represent 1 SE value

The developmental time and survival of the larval and pupal stages were significantly influenced by the larval food regimes. Adult body mass of females and males from the restricted larval food regime was markedly lower than those of adults from the high and variable larval food regimes (females, F_2, 42_ = 44.997, *p* < 0.001; males, F_2, 42_ = 21.273, *p* < 0.001; Fig. 1a), but adults from the variable food regime and high food regime had similar adult body weights (Tukey test, females, *p* = 0.684; males, *p* = 0.502; Fig. 1a). Further, the developmental time of the resulting phenotypes from the restricted larval food regime was about five days longer than that of those from the variable and high larval food regimes (F_2, 42_ = 191.950, *p* < 0.001; Fig. 1b), with again no statistical difference between the latter two phenotypes (Tukey test, *p* = 0.106; Fig. 1b). The survival of the resulting ladybeetles undergoing the restricted larval food regime was significantly lower than that of the other two phenotypes (*χ*^2^ = 17.412, df = 2, *p* < 0.001, Fig. 1c).

### Reproductive performance

**Fig. 2:**
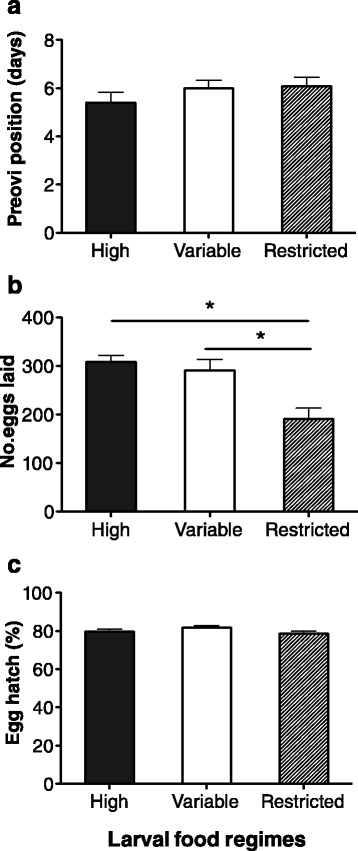
Reproductive performance of adult phenotypes resulting form high, variable and restricted larval food regimes. **a** Pre-oviposition period; **b**, Egg production; **c**, Egg hatch. Asterisks (*) indicate significant differences. Error bars represent 1 SE value

Egg production of the resulting adult phenotypes was significantly affected by larval food regimes. Although there was no difference in pre-oviposition period when the adults of all groups were provided with ad libitum foods from emergence on (Kruskal-Wallis test, *p* = 0.319; Fig. 2a), the egg production of the adult phenotypes from the restricted larval food regime was lower than that of adults from the high and variable larval food regimes (Kruskal-Wallis test, *p* < 0.001; Fig. 2b). However, females from the variable larval food regime produced the similar egg numbers as those from the high larval food regime (Fig. 2b). In contrast, there was no difference in egg hatch among the three phenotypes (F_2, 42_ = 1.640, *p* = 0.206; Fig. 2c).

### Starvation resistance

**Fig. 3:**
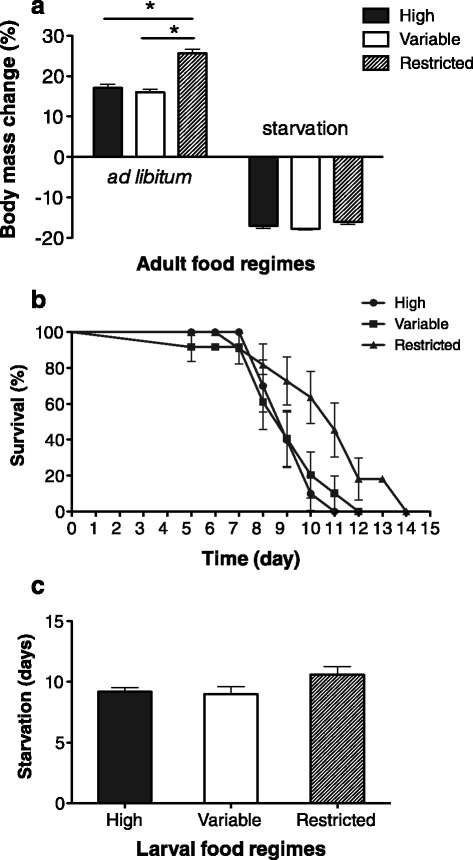
Resistance of adult phynotypes resulting from high, variable and restricted larval food regimes to a 5-day starvation period in the adult stage. **a** Body mass changes after having experienced the starvation period versus ad libitum food in the early adult stage; **b**, Survival curves of reproductively active adult phenotypes subjected to food starvation; **c**, Mean survival time from the start of food deprivation to death. Error bars represent 1 SE value

After experiencing a five-day period feeding on *P. citri* as larvae, the body mass increase of the females from the variable and high larval food regimes was lower than that of those which had experienced restricted food levels as larvae (F_2, 45_ = 52.894, *p* < 0.001; Fig. 3a). In contrast, no difference in body mass loss was observed among the treatment groups after female ladybeetles had experienced a five-day starvation period (F_2, 45_ = 2.373, *p* = 0.106; Fig. 3a). Further, the starvation resistance of reproductively active adults in terms of their survival time when given no food or water conditions did not differ among the three treatment groups (F_2, 27_ = 2.443, *p* = 0.106; Fig. 3b, c).

### Gene expression

**Fig. 4:**
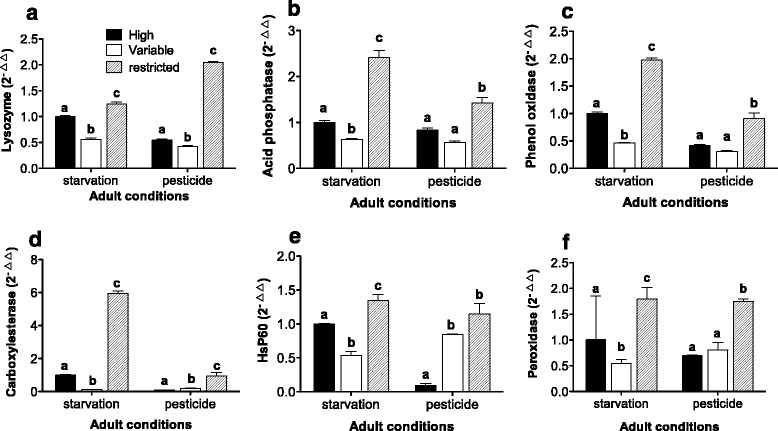
Expression of genes encoding immune-related and antioxidant-related enzymes in adult phenotypes resulting from high, variable and restricted larval food regimes after having been subjected to a 24 h starvation period or pesticide treatment in the adult stage. **a** Lysozyme; **b**, Acid phosphatase; **c**, Phenol oxidase; **d**, Carboxylesterase; **e**, HSP60; **f**, Peroxidase. Different letters within each treatment indicate significant differences. Error bars represent 1 SE value

The gene expression of female adults subjected to starvation or acetamiprid treatment was associated with the food regime they experienced as larvae. In both treatments, the adults from the restricted larval food regime had a higher expression of the selected genes than those from the high and variable larval food regimes (Fig. 4). In the starvation, the gene expression of the resulting phenotypes that had experienced the variable larval food regime was lower as compared with its two counterparts (Fig. 4).

## Discussion

Our findings indicate that the adult fitness of the ladybeetle *C. montrouzieri* is affected by larval nutritional conditions through developmental plasticity. The feeding regime of the larvae affected larval and pupal developmental time, with a longer time for insects experiencing the restricted larval food regime as compared with the variable or high larval food regimes, but with no difference between the latter two food regimes. The resulting adult phenotypes of *C. montrouzieri* also had a different body mass and reproductive output, and showed different expression levels of genes under stressful conditions in adult life.

The environmental conditions experienced by organisms during early life can exert significant effects on development, reproduction and physiological functions [2, 4, 31]. Food availability is an important factor influencing individual developmental rate [4, 21, 32]. Generally, when individuals grow under favorable conditions (e.g., abundant food, lack of predators, optimal climate) they acquire a fast growth rate and suffer low mortality; conversely, individuals are expected to show reduced growth rates and high mortality under unfavorable environmental conditions (e.g., food scarcity, extreme temperature, high predator pressure) [24]. The responses shown by *C. montrouzieri* to the tested range of larval food regimes indeed suggest that food availability significantly affected individual growth. The beetles experiencing the high larval food regime had a faster development and higher survival than those from the restricted larval food regime (Fig. 1b). Additionally, we found that *C. montrouzieri* larvae subjected to the variable larval food regime had a similar survival rate as those from the high larval food regime, implying that food supplements in the late larval stage reduce the risk of mortality. Several studies have reported that compensatory growth, which takes place when food availability improves after a period of food scarcity, is a common strategy in insects to limit reduction in adult body size and reproduction [4, 32, 33].

Various factors may affect individual reproductive performance including food availability and quality, mating success, population density and predation risks [23, 34, 35]. Among these factors, individual phenotypes (e.g., as characterized by body size) play an important role in determining reproduction [36]. Generally, in insects a large body size is associated with a high reproductive output, whereas small body size results in a low reproductive output under the same environmental conditions [32, 37, 38]. In our studies, we found that adults from the high larval food regime had greater body mass and higher egg production than those from the restricted food regime. Although ladybeetles from the variable larval food regime experienced nutritional restriction in the first eight days of larval development, they developed into adults with a similar body mass as those from high food regime, and had a similar egg production. This indicates that the compensatory growth took place in the later larval stages of *C. montrouzieri* after the switch in food supply, and that the larval nutritional conditions further affected adult fitness. Whereas previous studies have reported effects of body size on pre-oviposition period in other insect species [39, 40], no effects were found on pre-oviposition period or egg hatch in the present study (Fig. 2a). Whereas in our study there was no statistical difference in starvation resistance between the resulting adult phenotypes of *C. montrouzieri,* previous studies did find that starvation is likely to trade-off with other fitness-related traits (e.g. fecundity and lifespan) [24, 41].

We also observed that adults which had experienced the variable food regime as larvae displayed lower expression levels of genes encoding immune enzymes (i.e. lysozyme, acid phosphatase and phenol oxidase) than did those from the high and restricted food regime, after having experienced a short starvation period (24 h). Organismal immunity plays important roles in defending against harmful chemicals, pathogenic microorganisms, or parasites [42, 43]. Studies on *Drosophila melanogaster* have reported that the protein level of foods consumed by larvae may affect their immune activities [22]. Interestingly, the resulting phenotypes from the restricted larval food regime showed a higher gene expression of lysozyme, acid phosphatase and phenol oxidase (Fig. 4a, b, c) than those undergoing the high and variable larval food regime under conditions of stress (i.e. starvation or exposure to an insecticide). This result indicates that the proportion of larval resources allocated to immunity may be independent of its effect on the organism’s general condition (e.g., development and reproduction). That the expression level of immune genes can be affected by larval nutrition has also been reported in *Drosophila melanogaster* [22]. In the katydid, *Kawanaphila nartee* food availability has been noted to mediate the immune investment and reproductive efforts of females [44]. Generally, most resources will be first allocated to maintenance rather than reproduction and/or immune activities, especially under a food shortage condition [45].

The different phenotypes of *C. montrouzieri* resulting from larval feeding regimes also showed distinct antioxidant abilities as indicated by the expression of genes encoding antioxidant enzymes (i.e. peroxidase and HSP60), indicating different potentials of the nutrition-induced phenotypes in response to reactive oxygen species (ROS). ROS are the by-products of normal metabolic activities that may damage key biomolecules such as DNA, proteins and lipids [46]. Peroxidase and HSP60 play crucial roles in defending the organism against detrimental effects of the major ROS: superoxide anions and hydrogen peroxide [47]. As other two key antioxidant enzymes, SOD contributes to dismutating the superoxide anions into hydrogen peroxide and CAT further dismutates it into water [48].

Although developmental plasticity induced by nutritional conditions does not involve gene mutation or changes in DNA sequences, it is an important adaptive process in response to varying environmental conditions. The results of this and previous studies suggest that the developmental rate and body size of *C. montrouzieri* are largely dependent on larval food availability [21]. Arguably, many other factors (including temperature, humidity or photoperiod) may also exert impacts on its fitness and on the expression level of other genes encoding different functional proteins. The exact mechanisms and contributory factors involved in developmental plasticity are still not fully understood. Previous studies have noted that the organismal phenotypes result from the combination of external environment and gene inputs [1, 35]. The expected fitness of individuals who experience the developmental inputs and develop the phenotype should be higher than that of individuals who experience the developmental inputs but do not develop the phenotype [49]. External environment has been noted to affect the expression of genes by impacting on DNA methylation, protein modification and histone acetylation [1, 50]. This process may shield genetic variation from natural selection, which presumably promotes the accumulation of cryptic variation that does not result in phenotypic variation even though having encountered genetic variation [1, 2].

## Conclusions

*C. montrouzieri* may plastically respond to the variable nutritional conditions by altering its growth rate and allocation of resources, resulting in a variation of its body size and reproductive performance, as well as the expression of the immune- and antioxidant-related genes. Developmental plasticity is a crucial phenomenon, which is consequential for adaptability and diversity because the variation of individual phenotypes and resistance to stressful conditions may be associated with variation in developmental inputs in earlier larval life stages. This study provides further insight into the mechanisms of nutrition-induced plasticity in insects, but more detailed studies are warranted to explore the underlying mechanisms behind the different expression level of genes encoding immune- and antioxidant-related enzymes, and a further investigation is needed to examine whether the nutrition-induced phenotypes affect the fitness of future generations.

## Abbreviations

ANOVA: One-way analysis of variance
ROS: Reactive oxygen species
qPCR: Quantitative real-time PCR
SE: Standard error
HSP60: Heat shock protein 60
SOD: Superoxide dismutase
CAT: Catalase
